# Molecular Phylogenetics of the Genus *Neoconocephalus* (Orthoptera, Tettigoniidae) and the Evolution of Temperate Life Histories

**DOI:** 10.1371/journal.pone.0007203

**Published:** 2009-09-25

**Authors:** Robert L. Snyder, Katy H. Frederick-Hudson, Johannes Schul

**Affiliations:** Biological Sciences, University of Missouri, Columbia, Missouri, United States of America; McGill University, Canada

## Abstract

**Background:**

The katydid genus *Neoconocephalus* (25+ species) has a prominent acoustic communication system and occurs in large parts of the Neotropics and Nearctic. This group has been subject of numerous behavioral, physiological, and evolutionary studies of its acoustic communication system. Two distinct life histories occur in this group: The tropical life history incorporates multiple generations/year and direct egg development without environmental triggers. Temperate life history is characterized by overwintering in the egg stage, cold trigger of egg development, and one generation/year. This study reconstructs the phylogenetic relationships within the genus to (1) determine the evolutionary history of the temperate life history, and (2) to support comparative studies of evolutionary and physiological problems in this genus.

**Methodology/Principal Findings:**

We used Amplified Fragment Length Polymorphisms (AFLP), and sequences of two nuclear loci and one mitochondrial locus to reconstruct phylogenetic relationships. The analysis included 17 ingroup and two outgroup species. AFLP and mitochondrial data provided resolution at the species level while the two nuclear loci revealed only deeper nodes. The data sets were combined in a super-matrix to estimate a total evidence tree. Seven of the temperate species form a monophyletic group; however, three more temperate species were placed as siblings of tropical species.

**Conclusions/Significance:**

Our analyses support the reliability of the current taxonomic treatment of the *Neoconocephalus* fauna of Caribbean, Central, and North America. Ancestral state reconstruction of life history traits was not conclusive, however at least four transitions between life histories occurred among our sample of species. The proposed phylogeny will strengthen conclusions from comparative work in this group.

## Introduction

The katydid genus *Neoconocephalus* (Karny 1907) is characterized by loud calls that can be heard by the human ear for long distances [1]. This group has been used to study many aspects of acoustic communication, such as chorusing behavior [2], [3], acoustic spacing [4], [5], mechanics [6] and energetics of calling [7]–[9], developmental plasticity of calling behavior [10], [11], recognition of conspecific calls during phonotaxis [12]–[15], and sexual selection [16], [17]. Many of these studies have been conducted comparatively among several *Neoconocephalus* species; however, their power has been limited without knowledge of the phylogenetic relationships within this genus.

Systematics and biogeography of *Neoconocephalus* was reviewed in detail [18]. *Neoconocephalus* species occur in grasslands in Neotropical and Nearctic regions. Twelve species of *Neoconocephalus* are limited to the Nearctic (Table 1) and have life histories typical of temperate katydids: they have one reproductive generation per year (univoltine) and overwinter as diapausing eggs [19]. Eggs do not start developing until after they are exposed to a period of cold temperatures (JS, pers. observ.). All but two of these species have wide ranges in North America. Only *N. lyristes* is limited to two disjunct populations at the Atlantic coast and the Great Lakes, and *N. pahayokee* is endemic to the Florida Everglades [19], [20].

**Table 1:** The 25 species of *Neoconocephalus* with ranges in North America, Central America and the Caribbean.

	Range	Life history	Sources
*N. aduncus* (Scudder 1879)	Cuba	tropical	2
***N. affinis*** (Beauvois 1805)	throughout Caribbean & Central America	tropical*	2, 3, 4
***N. bivocatus*** Walker Whitesell, Alexander 1973	Eastern USA	temperate*	1, 4
N. carbonarius (Redtenbacher 1891)	Cuba, Grand Cayman	tropical	2
***N. caudellianus*** (Davis 1805)	South Eastern USA	temperate	1, 4
***N. ensiger*** (Harris 1841)	Eastern USA	temperate*	1, 4
***N. exciliscanorus*** (Davis 1887)	Eastern USA	temperate*	1, 4
N. lyristes (Rehn and Hebard 1905)	USA: Great Lakes region, New Jersey	temperate	1
***N. maxillosus*** (Fabricius 1775)	throughout Carribbean	tropical*	2, 4
***N. melanorhinus*** (Rehn and Hebard 1907)	USA: Gulf coast, Atlantic coast	temperate	1
***N. nebrascensis*** (Bruner 1891)	Eastern USA	temperate*	1, 4
N. occidentalis (Saussure 1859)	Hispaniola	tropical	2
N. pahayokee Walker and Whitesell 1978	USA: Everglades (Florida)	temperate	1
***N. palustris*** (Blatchley 1893)	Eastern USA	temperate*	1, 4
N. pinicola Walker and Greenfield 1983	Hispaniola	tropical	2
N. pipulus Walker and Greenfield 1983	Jamaica	tropical	2
***N. punctipes*** (Redtenbacher 1891)	Central America, Trinidad, Jamaica	tropical	2,3,4
***N. retusiformis*** Walker and Greenfield 1983	Puerto Rico, Mona	tropical	1
***N. retusus*** (Scudder 1878)	Eastern USA	temperate*	1, 4
***N. robustus*** (Scudder 1862)	Eastern USA, California	temperate*	1, 4
***N. saturatus*** (Griffini 1899)	Tinidad, Grenada, St Vincent	tropical	2
***N. spiza*** Walker and Greenfield 1983	Central America	tropical	2, 3, 4
N. susurrator Walker and Greenfield 1983	Trinidad	tropical	2
***N. triops*** (Linnaeus 1758)	South, Central, North Am., Caribbean	tropical*	1, 2, 3, 4
***N. velox*** (Rehn and Hebbard 1914)	South Eastern USA	temperate	1

Species in bold are included in our phylogenetic analysis. Life history is given after Greenfield (1990) or as observed by the authors (asterisks). Temperate life history is characterized univoltism and overwintering in the egg stage (eggs develop only after cold treatment). Tropical life history is characterized by direct egg development and multivoltism. Sources: 1 [25] 2 [22] 3 [47] 4 own observations.

Three species with predominantly tropical distribution (*N. triops, N. affinis, N. maxillosus*) occur in North America. *N. triops* is widespread in the southern USA reaching as far north as Ohio [21] and Missouri (JS, pers. observ.), while *N. affinis and N. maxillosus* are limited to the southern parts of Florida [22], [23]. These three species have life histories typical of tropical katydids with several reproductive generations per year and egg development starting directly after oviposition without an environmental trigger[18].

The taxonomic treatment of the North American *Neoconocephalus* is well established. Most species were described by 1915 [18], and after the work of Walker and collaborators in the 1960s and 70s [19], [20], [24], [25], there is little doubt that the 15 species mentioned above constitute the North American *Neoconocephalus* fauna. Unfortunately, the situation for the neotropical *Neoconocephalus* is much more complicated. More than 100 species were named by 19th and early 20th century cataloguers [26]. The reliability of these names is highly doubtful, as evidenced by the treatment of *N. triops*: at least 14 synonyms are recognized today [22], [26], with 4 of them described in one monograph [27].

Walker and Greenfield [26] revised the taxonomy of *Neoconocephalus* from the Caribbean and Panama, recognizing 13 species, including 5 new species (3 of these 13 species also occur in North America). However, 36 *Neoconocephalus* species names had been proposed prior to 1900, indicating that the early taxonomic work is not reliable. Three of the 13 recognized [26] species (*N. triops, N. affinis, N. maxillosus*) are widespread in the Caribbean and adjoining continental mainlands. The other nine are known only from restricted continental ranges and nearby islands or from one or a few Caribbean islands (Table 1).

Phylogenetic relationships within this genus are largely unclear. In 1915 a phylogeny based on morphological characters was proposed [28]. This phylogeny has been used until recently [18] to propose evolutionary hypotheses on aspects of the communication system. Since the genus *Neoconocephalus* has been intensively used to study evolutionary questions, a well substantiated hypothesis about the phylogenetic relationships within this genus will add significantly to the value of comparative studies and the evolutionary inferences drawn.

In this study, we reconstruct the phylogenetic relationships within the genus *Neoconocephalus* to provide a tool for evolutionary and comparative studies in this group and to determine if temperate life history traits evolved once or multiple times among North American *Neoconocephalus*. The closest relative of *Neoconocephalus* is undoubtedly the old world genus *Ruspolia* (Schulthess Schindler 1898) [18]. It has been proposed that *Neoconocephalus* might be the result of a dispersal event from Africa to tropical South America, as evidenced by individuals of *Ruspolia* that landed on ships 1000 km off the African coast [29]. This scenario suggests that the tropical life history represents the ancestral state in *Neoconocephalus*, and that temperate life history traits evolved subsequently as *Neoconocephalus* spread into North America. We test this prediction and estimate the number of transitions between tropical and temperate life histories in our sample of *Neoconocephalus* species.

## Materials and Methods

### Taxon sampling

We restricted our phylogenetic analysis to the well-established *Neoconocephalus* fauna of the Caribbean, and Central and North America, as reliable information on the South American *Neoconocephalus* fauna is not available (see introduction). We included in our sampling all Caribbean, North and Central American species with wide distributions: the missing eight species from this range are either endemic to one or a few Caribbean islands (*N. aduncus, N. carbonarius, N. occidentalis, N. pinicola, N. pipulus, N. susurrator*) [22] or have limited continental ranges (*N. lyristes, N. pahayokee*) [28] (Table 1). Specimens of *Belocephalus davisi* Rhen and Hebard, 1916 and *Bucrates malivolans* (Scudder 1878) were used as outgroups in the phylogenetic anaylses. Both *Belocephalus* and *Bucrates* are considered close relatives of *Neoconocephalus* [25] and are grouped in the same tribe (Copiphorini) [26]. Specimens of *Ruspolia*, the presumed sibling of *Neoconocephalus* [18], were not available for our analysis. All taxa included in our phylogenetic analysis were unequivocally identified using the criteria given in [22], [25]. The vouchers of all specimens are kept in the collection of JS.

Genomic DNA was isolated from ethanol-preserved hind femurs using a DNeasy Blood + Tissue Kit (Qiagen Inc., Valencia, CA, USA). The concentration and quality of each DNA sample were determined by spectrophotometry (NanoDrop 1000, Thermo Scientific, Wilmington, DE, USA).

### Molecular markers

#### AFLP

Amplified Fragment Length Polymorphism (AFLP) banding patterns were generated using a modified version of the protocol described in [30], that allowed for fluorescent detection of labeled AFLP's. Genomic DNA was digested with the restriction enzymes EcoR I (Eco) and Mse I (Mse) (NEB, Ipswitch, MA, USA). Synthetic double stranded DNA adaptors were ligated to the overhanging ends of the respective restriction sites (Eco and Mse). Preselective PCR, using 100 fold dilute digestion/ligation product and the Eco+A (5′-GACTGCGTACCAATTCA-3′) and Mse+A (5′-GATGAGTCCTGAGTAAA-3′) primers (IDT, Coralville, IA, USA) were thermocycled at 72°C for 2 min, followed by a 94°C for 30 sec denature, 56°C for 30 sec anneal and 72°C for 2 min extension, repeated 30x, with a 60°C for 10 min hold. Selective PCR of 100 fold dilute preselective PCR product, used one of two labeled Eco primers either Eco+AAC (6FAM) or Eco+AGC (PET) (ABI, Foster City, CA, USA) and one of five Mse primers, either Mse+ATA, Mse+AGA, Mse+AGC, Mse+ACA or Mse+AAC. Primer-sets were amplified in separate PCR reactions, using concentrations of labeled and unlabeled primer of 0.04 µM and 0.2 µM, respectively, and the following thermocycling conditions; 94°C for 2 min, 94°C for 30 sec denature, 65°C reduced by 0.7°C/cycle (with a 1°/s ramp rate) annealing, 72°C for 2 min extension, repeated 12x, followed second cycle of 94°C for 30 sec, 56°C for 30 sec, and 72°C for 2 min, repeated 23x, with 60°C 30 min final hold. Individual selective PCR products were multiplexed and diluted, which resulted in a 1∶10 dilute of each PCR product. Samples were genotyped at the DNA Core Facility, University of Missouri, on an ABI 3730 genetic analyzer, with Liz600 internal size standard (Applied Biosystems, Foster City, CA, USA).

AFLP genotypes were analyzed in GeneMarker v1.6 (Softgenetic Corp, State College, PA, USA) using the AFLP analysis setting.

#### Gene trees

Partial gene sequences were generated for 2 nuclear loci and 1 mitochondrial locus; Internal Transcribed Spacer 1 and 2 (ITS1 & ITS2), the protein coding Histone 3 (H3) and mitochondrial Cytochrome Oxidase I (COI). The data from ITS1 and ITS2 was combined into a single ITS analysis. The ITS loci are separated by the 5.8S rRNA gene, which is less than 1000 bp long. Given the close proximity of the two loci we assume them to be tightly linked thus treat them as a single locus.

PCR amplification was performed on an Eppendorf Mastercycler gradient (Eppendorf-Brinkman Instruments Inc., Westbury NY, USA) using *Taq* DNA polymerase (GoTaq, Promega Corp. Madison, WI, USA). ITS1 PCR primers were anchored in the flanking 18s and 5.8s genes and ITS2 PCR primers were anchored in the flanking 5.8s and 28s genes. Primer sequences for both ITS genes correspond to the CAS18sF1 (ITS1 forward), CAS5p8sB1d (ITS1 reverse), CAS5p8sFc (ITS2 forward) and CAS28sB1d (ITS2 reverse) of [31]. H3 primers (H3 AF and H3 AR) sequences were from [32]. COI primers (Ron and Calvin) were from [33]. All primers were used at a concentration of 10 µM. Thermocycling conditions for all three primer-sets are as follows: Denaturation at 94°C 50 sec, annealing at 50°C 1 min (COI), or 51°C 1 min (H3 & ITS2), or 56°C 1 min (ITS1), extension 72°C 1 min, repeated 35x, with a final 72°C extension for 6 min. Amplified PCR products were prepared for sequencing using an enzymatic clean-up (1∶1 ratio of Exonuclease I and Antarctic Phosphotase, New England BioLabs Inc., Ipswitch, MA, USA) in a 1∶10 reaction of enzymes to PCR product and incubated at 37°C for 50 min. Sequencing was performed at either the DNA Core Facility, University of Missouri, Columbia, MO or at Cornell Life Sciences Core Laboratories Center, Cornell University, Ithaca, NY, on ABI 3730 DNA Analyzers, using standard Big Dye Terminator cycle sequencing chemistry (Applied Biosystems, Foster City, CA, USA). Resulting sequence data was edited and aligned in Sequencher v4.5 (Gene Codes Corp., Ann Arbor, MI, USA).

All sequence and AFLP data are available at GenBank (Accession Numbers FJ913499-FJ913766).

### Phylogenetic Analysis

#### AFLP

The AFLP character matrix was analyzed using either Bayesian or distance based optimality criteria. Distance analysis used a Nei & Li model [34] of genetic distance and bootstrapping to measure nodal support, and was performed in PAUP* 4beta v.10 [35]. A Bayesian approach does not restrict tree searches to the most parsimonious trees and allows an evolutionary model to be incorporated. The MCMC settings for each MrBayes [36] analysis were 4 runs, of 10 chains each, for 2 million generations. Runs were determined to have reached a stationary distribution based on split frequencies reported in MrBayes and by plotting the log likelihood values of the cold chain. The MCMC runs were sampled every 100 generations, resulting in 20,000 trees per run. Posterior probabilities were calculated after a ‘burnin’ of 5000 trees, from four independent MrBayes analyses. Because AFLP data reports the presence or absence of characters, we used a restriction site model for each analysis. The restriction site model was binary, similar to the F81 models of [37], and used the ‘noabsencesite’ sub-model of MrBayes. The noabsencesite sub-model best fits the nature of AFLP's, because the DNA fragments are anonymous and analysis cannot distinguish between the absence of an allele and the absence of that locus [38].

#### Gene trees

*G*ene sequences were analyzed in both separate and combined analyses [39]. Phylogenetic trees were inferred using Bayesian optimality criterion implemented in MrBayes v3.1.2 [39] and computed on the computer cluster at the Computational Biology Service Unit at Cornell University (Ithaca, NY). Models of nucleotide substitution were selected using an Akaike Information Criterion (AIC) in MrModeltest 2.2 [40]. The MCMC settings for each MrBayes analysis were: 2 runs, 10 chains each, for 2 million generations. Each MrBayes analysis was run three times independently to ensure that each run achieved similar stationary likelihood values (cold chain in stationary phase). Each run was considered to have reached a stationary distribution based on split frequencies reported in MrBayes and by plotting the log likelihood values of the cold chain. The MCMC runs were sampled every 100 generations, resulting in 20,000 trees per run. The first 5000 trees of each Bayesian run were discarded as burnin, and the remaining trees in each analysis were used to calculate the posterior probabilities and 50 % majority rule consensus tree.

#### Total evidence tree

The three gene alignments and AFLP data were concatenated into a super matrix of 4089 characters and all individuals (128) in this study. This approach allowed us to preserve the alignments of individual data partitions. AFLP data were available for 124 individuals and data for the three gene sequences were available for 67 individuals (see Table S1). Data for missing data partitions (i.e. AFLP or gene sequences) of an individual were coded as question marks. For analysis in MrBayes, models and priors were identical to those used to build each gene tree and were unlinked between all partitions (genes, AFLP). This tree was produced in the same manner as the gene trees (2 runs, 10 chains each, 2 million generations). The first 5000 trees of each Bayesian run were discarded as burnin, and the remaining trees in each analysis were used to calculate the posterior probabilities and 50 % majority rule consensus tree.

### Ancestral Character State Reconstructions

The character state for the life history (tropical or temperate) was reconstructed on the total evidence consensus tree using parsimony character tracing (MacClade) [41]. In addition, character states were calculated for the same tree (using all 30,000 post burnin trees) with Bayesian methods in BayesTraits [42] (30 million generations, 3 runs each, 50,000 burnin generations). Statistical support for the Bayesian ancestral state reconstruction was determined using posterior probabilities which were calculated with the ‘MRCA’ command in BayesTraits [42]. Support of the reconstructed ancestral state was generated using a log Bayes Factor test. The log Bayes Factor was calculated from the harmonic means calculated with the ‘fossil’ command in BayesTraits [42]. Character state reconstruction is considered strongly supported if the log Bayes Factor is greater than 6 [42].

## Results

### AFLP

**Figure 1:**
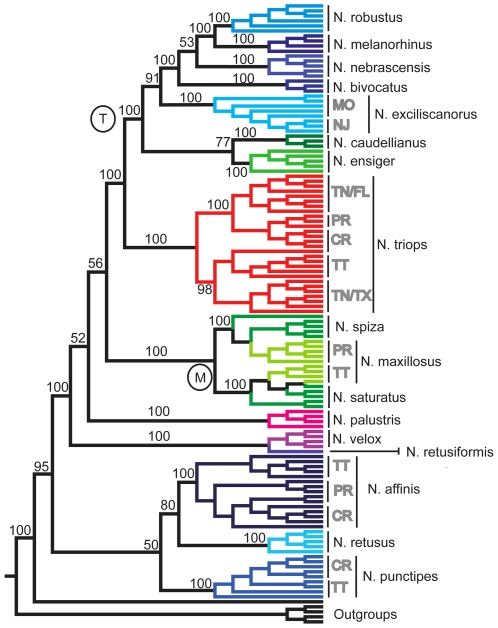
Phylogeny of *Neoconocephalus* based on AFLP analysis. Majority rule Bayesian AFLP phylogeny of 17 species of katydids. The species, as identified after [22], [25] are indicated by color coding. Five species are represented by multiple geographic populations; CR = Costa Rica, FL = Florida, USA, NJ = New Jersey, USA, MO = Missouri, USA, PR = Puerto Rico, TN = Tennessee, USA, TT = Trinidad, and TX = Texas, USA. Nodal Support values, reported above nodes, are posterior probabilities in percent. Asterisks at the species names indicates temperate life history. The individuals included in this analysis are listed in the Table S1 in the sequence as they appear in this tree.

Our phylogenetic analysis included 119 individuals of the ingroup and 5 of the outgroup (see Table S1) and utilized 1680 polymorphic AFLP characters (out of 2028 total characters). The resulting data matrix yielded similar phylogenetic topologies using both distance and Bayesian analyses. The majority-rule consensus tree of the Bayesian analysis is given in Figure 1. All but 3 ingroup species formed monophyletic clades, even when sampled from distant localities. The 3 species of the ‘*N. maxillosus* clade’ (label M in Figure 1) were not resolved (see below). The species with tropical life history (i.e. direct egg development) branch off basally in the phylogeny, while 7 of the 10 species with temperate life history, form one monophyletic clade (label T). Sibling to this temperate clade was *N. triops*, the only tropical species with a range that extends into temperate North America [21]. Several basal nodes of the tree had low posterior probabilities; however, these lineages include six species with tropical life history and three temperate species with egg diapause (*N. velox, N. palustris, N. retusus*).

We sampled two populations of *N. maxillosus*. Individuals from Trinidad grouped with *N. saturatus* from the same island, while individuals from Puerto Rico grouped with *N. spiza* collected in Costa Rica. This was not due to errors in species identification, as male calls of *N. maxillosus* are distinctly different from those of the other two species, and all samples of the ‘*N. maxillosus* clade’ were males collected while calling (see discussion).

### Nuclear genes H3 and ITS

The phylogenetic analyses based on H3 and ITS contained 64 ingroup and 3 outgroup taxa (see Table S1). MrModeltest selected the K80 model of nucleotide substitution for the ITS data set and the GTR+I+G models for the H3 dataset. The ITS data set resulted in 878 aligned base pairs and 106 parsimony informative characters, while the H3 data set resulted in 308 aligned base pairs and 24 parsimony informative characters.

**Figure 2 pone-0007203-g002:**
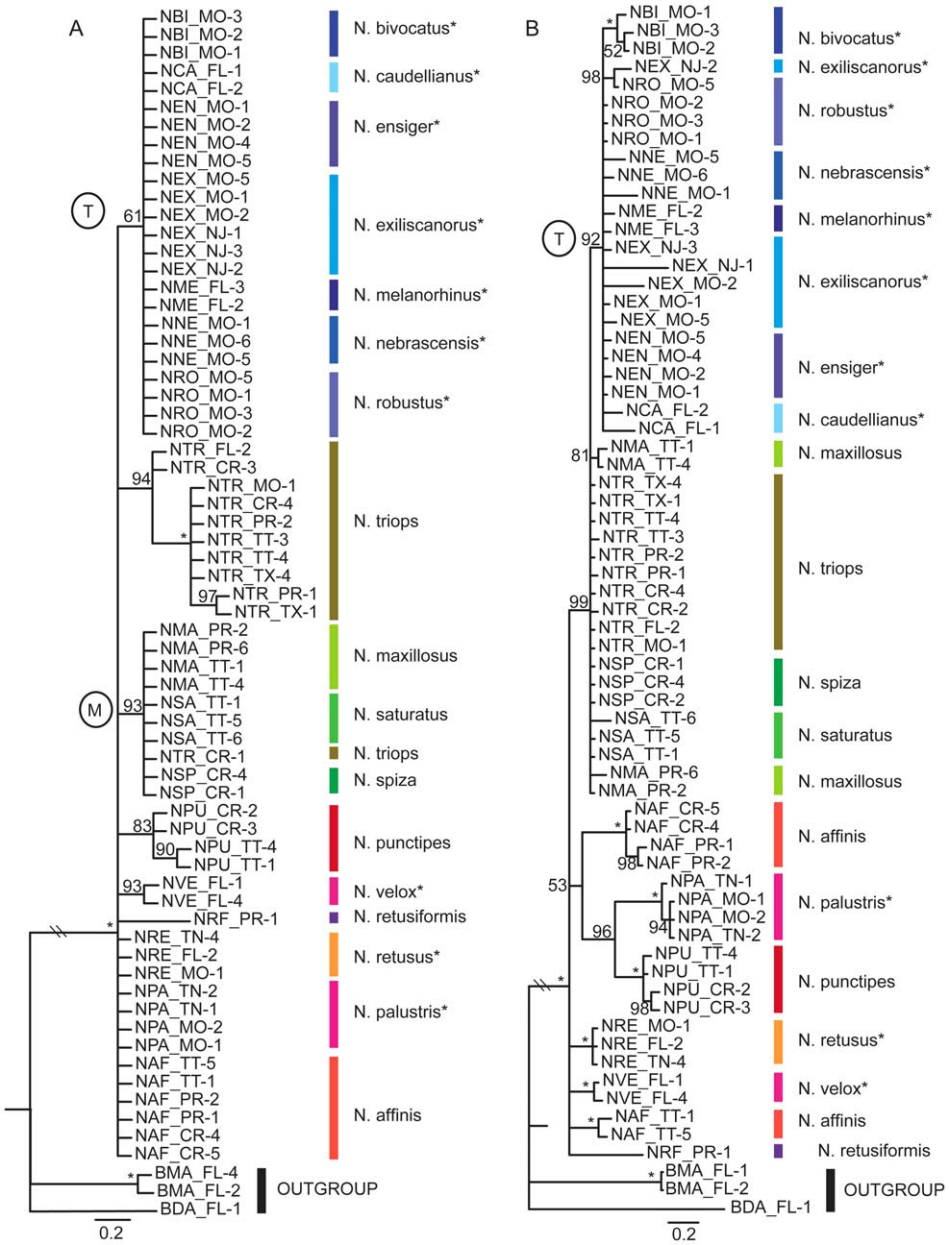
Phylogeny of *Neoconocephalus* based on nuclear genes. Bayesian phylogeny of *Neoconocephalus* using partial nDNA sequences H3 (a) and ITS (b). Nodal support values given as posterior probabilities in percent; asterisk indicate 100%. The temperate clade and the *N. maxillosus* clade are indicated by labels ‘T’ and ‘M’, respectively. Branch lengths drawn to scale. The taxa included in these phylogenies are listed in Table S1. Asterisks at the species names indicates temperate life history. Species are indicated by colored lines.

Phylogenies based on either nuclear gene provided little resolution at the species level (Figure 2a, b). Both phylogenies grouped the seven species of the ‘temperate clade’ in the AFLP tree together in one separate clade (label T in Figure 2). Also, *N. retusus, N. velox*, and *N. palustris* appeared among tropical species in both nuclear phylogenies.

The H3 phylogeny grouped *N. maxillosus, N. saturatus*, and *N. spiza* together (label M, Figure 2a). In the ITS tree, these three species grouped together with *N. triops* and the temperate clade, which was congruent with their position in the AFLP tree. Finally, *N. affinis* was polyphyletic in the ITS tree: individuals from Trinidad group separately from *N. affinis* from Costa Rica and Puerto Rico (Figure 2b).

### Mitochondrial gene COI

The COI tree contained 64 ingroup and 3 outgroup taxa (table 1). DNA sequencing resulted in 875 aligned base pairs and 287 parsimony informative characters. The tree was constructed using a GTR+I+G models of nucleotide substitution.

**Figure 3 pone-0007203-g003:**
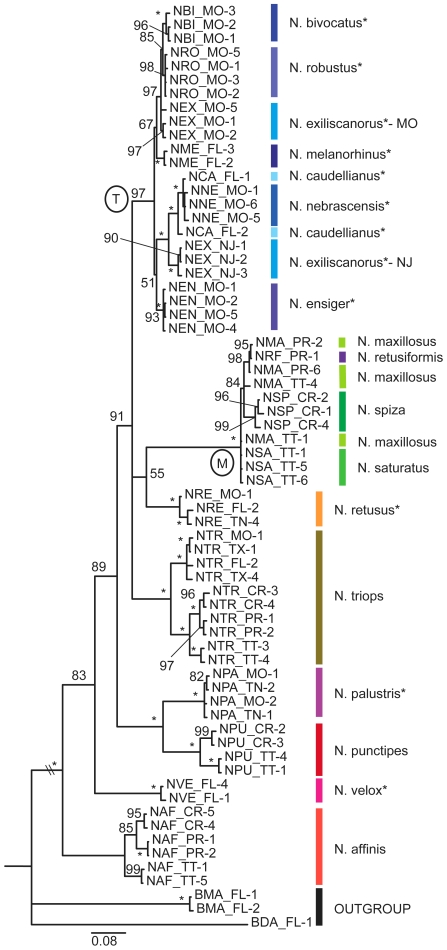
Phylogeny of *Neoconocephalus* based on a mitochondrial gene. Bayesian phylogeny of *Neoconocephalus* using partial COI mtDNA sequences. Nodal support values are posterior probabilities in percent; asterisk indicate 100%. The temperate clade and the *N. maxillosus* clade are indicated by labels ‘T’ and ‘M’, respectively. Branch lengths drawn to scale. The taxa included in this tree are listed in Table S1. Asterisks at the species names indicates temperate life history. Species are indicated by colored lines.

The COI phylogeny was well supported and most nodes were resolved with strong statistical support (Figure 3). As in the previously shown trees, the temperate clade of seven species was well supported by the COI data set. Similarly, the *N. maxillosus*-group and *N. triops* were close relatives of the temperate clade. However, *N. retusiformis* (collected in Puerto Rico) was grouped among the *N. maxillosus* from Puerto Rico. *N. retusus* grouped as the sibling to the *N. maxillosus*-group. The two other temperate species, *N. velox*, and *N. palustris* grouped, as in the previously described trees, with the tropical species.

Two populations of *N. exiliscanorus* had divergent mitochondrial haplotypes. The Missouri population grouped with *N. robustus* and *N. bivocatus*, both also collected in Missouri. The *N. exciliscanorus* population from New Jersey grouped with *N. velox* and *N. nebrascensis*.

### Combined analysis of ITS, H3, and COI

**Figure 4:**
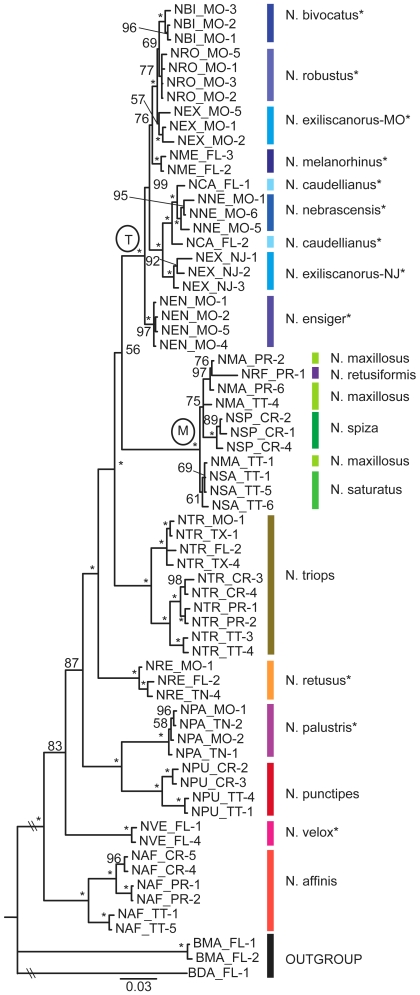
Phylogeny of *Neoconocephalus* based on the combined analysis of the nuclear and mitochondrial genes. Bayesian phylogeny of *Neoconocephalus* using the combined H3, ITS and COI data sets. Nodal support values are given as posterior probabilities in percent; asterisk indicate 100%. The temperate clade and the *N. maxillosus* clade are indicated by labels ‘T’ and ‘M’, respectively. Branch lengths drawn to scale. The taxa included in this tree are listed in Table S1. Asterisks at the species names indicates temperate life history. Species are indicated by colored lines.

The analysis of the combined ITS, H3, and COI data set was dominated by the 287 parsimony informative characters in the COI data set which accounted for nearly 70% of the 417 informative characters in the 3 gene combined analysis. The Bayesian phylogeny of the combined data sets is given in Figure 4.

The combined analysis corroborated the monophyly of a temperate group of 7 species. It placed the tropical *N. maxillosus* group (including *N. retusiformis*) as sibling to the temperate groups, with *N. triops* branching off basally to these groups. However, the support for this branch was low (0.56), leaving the position of these three clades (temperate clade, *N. maxillosus*-clade, *N. triops*) questionable. The 3 other temperate species (*N. retusus, N. velox, N. palustris*) were distributed among the tropical species. As in the COI tree, the combined analysis showed *N. exciliscanorus* as polyphyletic within the temperate clade.

### Total evidence tree

**Figure 5.Total:**
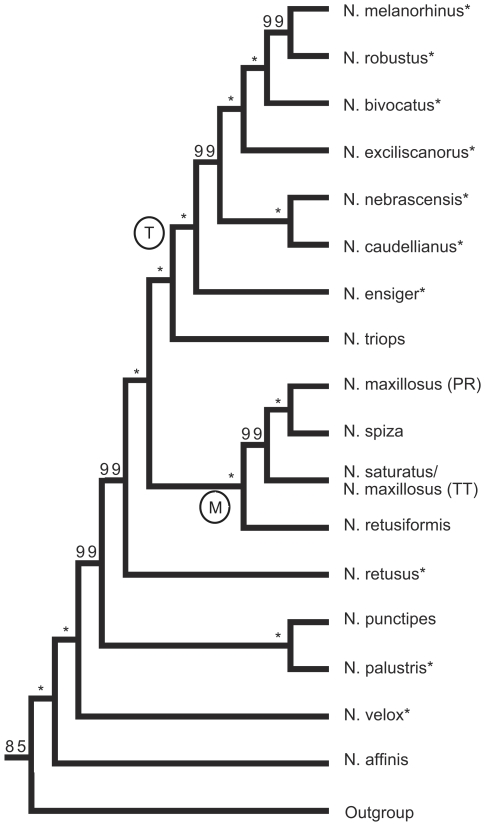
evidence tree of *Neoconocephalus*. Bayesian phylogeny of 17 *Neoconocephalus* species based on the combined analysis of the AFLP and gene trees given in Figures 1, 2, and 3. The phylogeny was pruned for visual simplification. Each tip of the pruned phylogeny represents a monophyletic group. Nodal support values are given as posterior probabilities in percent; asterisk indicate 100%. The temperate clade and the *N. maxillosus* clade are indicated by labels ‘T’ and ‘M’, respectively. Branch lengths are not drawn to scale. Asterisks at the species names indicates temperate life history.

We present the total evidence tree, which combines the AFLP tree and the three gene trees (Figures 1–3) in Figure 5. Individuals of each species (except *N. maxillosus*) grouped together in distinct clades, even when collected from different locales. Nodes to species, as well as more basal nodes were all extremely well supported (posterior probabilities above 0.98).

The clade of 7 temperate species (label T) appeared again in the total evidence tree. Within this clade, *N. nebrascensis* and *N. caudellianus* grouped as sibling species. The *N. maxillosus* clade (label M) contained in the total evidence tree *N. retusiformis*. As in all individual trees, *N. maxillosus* remained polyphyletic: individuals from Puerto Rico were the sibling clade to *N. spiza*. while *N. maxillosus* from Trinidad formed one clade with *N. saturatus* from the same island. Three species with temperate life history (*N. velox, N. retusus, N. palustris*) were distributed among the tropical species.

### Ancestral state reconstruction: Temperate and Tropical Life history traits

We estimated the ancestral state for life history (temperate with egg diapause vs. tropical with direct egg development, Tab. 1) on the topology of the total evidence tree (Figure 5). We assigned the character state of the outgroup as ‘uncertain’. Using parsimony, both character states (tropical/temperate) are equally likely to be the ancestral state in *Neoconocephalus* and require four character changes. The Bayesian method also failed to produce conclusive results: the tropical life history had somewhat higher posterior probability to be the ancestral state (0.58) than the temperate state (0.41), but this was not well supported in the log Bayes factor test (logBF = 1.03; logBF >6 are considered strong support [42]).

## Discussion

We estimated the phylogeny of the genus *Neoconocephalus* using several molecular markers. Both the AFLP and the COI data provided resolution at the species level. Two nuclear loci (H3, ITS) did not provide resolution of the species, but did resolve deeper nodes. The four phylogenetic trees were largely congruent with only minor discrepancies. Accordingly, the total evidence tree was extremely well supported, and all but one species were monophyletic in this tree. This supports the reliability of the current taxonomic treatment of the *Neoconocephalus* of the Caribbean and Central and North America as outlined in [18].

In the AFLP tree, *N. exciliscanorus* was monophyletic, while in the COI tree two populations appeared in separate positions: specimens from New Jersey grouped with the *N. nebrascensis/N. caudellianus* clade, while the Missouri population was sibling to *N. robustus/N. bivocatus*. This is likely an artifact due to an inherent problem with mitochondrial trees. Mitochondrial capture, e.g. through hybridization, and subsequent fixation of the captured mitochondrial haplotype may result in misleading phylogenetic information [43], [44]. The total evidence tree supported the conclusions from the AFLP analysis and a monophyletic species in the temperate clade. Extant ranges of *N. exciliscanorus* overlap extensively with *N. robustus, N. caudellianus*, and *N. nebrascenesis*, and at our collection sites of *N. exciliscanorus* we also found *N. robustus* as well as *N. nebrascensis* (Missouri) or *N. caudellianus* (New Jersey) in close vicinity.

Throughout our analysis, *N. maxillosus* was polyphyletic. The Puerto Rico population grouped with *N. spiza* (collected in Costa Rica) in the AFLP, gene, and total evidence tree (Figures 1, 4, 5). The Trinidad population grouped with *N. saturatus* from the same island in the AFLP and total evidence tree, however in the combined gene tree (Figure 4) one Trinidad individual grouped with the Puerto Rico individuals, another with *N. saturatus*. The calls of *N. maxillosus* are continuous, while both *N. spiza* and *N. saturatus* produce short chirps repeated at 1–5 chirps/s [22]. All individuals of *N. saturatus and N. maxillosus* from Trinidad were males (Tab. 2) collected while calling. In addition, *N. maxillosus* is morphologically distinct from the sympatrically occurring *N. saturatus*, so the misidentification of the species is extremely unlikely. The grouping of the Trinidad population of *N. maxillosus* with *N. saturatus* therefore suggests gene flow between these two species in Trinidad and/or South America. The alternative explanation of convergent evolution of these two populations appears unlikely as the Puerto Rico and Trinidad *N. maxillosus* were uniform in seemingly unrelated morphological and call traits. Also the placement of the Trinidad individuals in the gene tree does not support this alternative.

### Call traits and ranges of siblings

Male *Neoconocephalus* produce species specific calls which females use to localize and approach males for mating [18]. Typically, among co-occurring species calls differ distinctly in their temporal pattern, which allows females to selectively approach conspecific males [14], [22], [45]. Species with similar temporal patterns are typically not found signaling at the same time and place.

The total evidence tree (Figure 5) suggests several pairs of sibling species with similar calls but separate distributions. For example, *N. robustus* and *N. melanorhinus* both produce continuous calls with fast pulse rates. *N. melanorhinus* is a habitat specialist which occurs only in salt marshes, while *N. robustus* is widespread in grasslands of the eastern USA [25]. Both *N. nebrascensis* and *N. caudellianus* produce discontinuous calls with similar temporal pattern. The ranges of the two species overlap only marginally, with *N. caudellianus* being limited to the south-eastern USA and *N. nebrascensis* occurring in the north-central USA [25]. The two sibling species *N. palustris* and *N. punctipes* are very similar in body size as well as in spectral and temporal call characteristics [22], [25]. *N. punctipes* has tropical life history and occurs in Central America, while *N. palustris* has temperate life history and occurs in North America (Table 1).

In contrast, there was at least one clade with sympatric species, that differ notably in call traits. The ranges of *N. robusts, N. bivocatus*, and *N. exciliscanorus* overlap largely [25], and the three species commonly communicate within hearing distance of each other (JS, KFH, personal observations). The calls differ markedly from each other, with *N. bivocatus* having a different pulse pattern from the two other species [13], and *N. exciliscanorus* having a discontinuous call in contrast to the continuous calls of the two other species [18].

The pattern of allopatric siblings with similar call patterns suggests that ecological factors may have driven the diversification of *Neoconocephalus*. However, the sympatric clade with different call patterns suggests that at least in some cases the communication system played a role in the diversification of this group. Whether novel call patterns initiated diversification and ultimately speciation, or whether the changes of the communication system followed ecological diversification (e.g. through reinforcement [46]) is at this time not clear. Large comparative studies of male calls and female call recognition in the framework of the phylogeny presented here are required to address this question.

### Life History

The ancestral state reconstruction of the life history traits based in the total evidence tree did not provide an answer regarding the ancestral life history of *Neoconocephalus*. However, the total evidence tree provided strong evidence that life history traits have changed multiple times independently within our sample of species. Thus, life history traits appear to be evolutionarily plastic, which should facilitate the transitions from tropical to temperate habitats (or vice versa).

We restricted our analysis to the well described *Neoconocephalus* fauna of the Caribbean, and Central and North America. Almost certainly, more *Neoconocephalus* species exist in South America, but no reliable taxonomic information is available [18]. The range of *N. triops*, a species with tropical life history, reaches from temperate regions of North America, through the Neotropics into the temperate regions of South America [26]. Likely, additional species with temperate life history are present in the temperate regions of South America.

Assuming that the initial dispersal of *Ruspolia/Neoconocephalus* to the new world took place from tropical (West)-Africa to tropical South America, it appears likely that the tropical life history represents the ancestral state. Accordingly, during the dispersal into North America temperate life history traits would have evolved multiple times. The ‘temperate clade’ of seven North American *Neoconocephalus* species suggests an adaptive radiation following the evolution of the temperate life history. The species in this clade occur in diverse habitats including prairies (*N. bivocatus*), forest edges (*N. nebrascensis*), freshwater marshes (*N. exciliscanorus*), and salt marshes (*N. melanorhinus*) [19], [25]. The species of this clade are also diverse in their calls, encompassing the range of temporal and spectral call parameters of all described *Neoconocephalus* calls [18], [25].

However, the data presented here neither support nor rejects the tropical life history as ancestral state of *Neoconocephalus*. To answer this question future phylogenetic studies will have to include the South American *Neoconocephalus* fauna as well as temperate and tropical species of the old world genus *Ruspolia*.

Our proposed phylogeny of the genus *Neoconocephalus* raised several ecological and evolutionary questions. It will inform comparative studies regarding the function and evolution of the acoustic communication system. *Neoconocephalus* has served as a model to study the evolution of male calls and female preferences. The phylogenetic background presented here will strengthen conclusions from such studies. Due to the large body of data available for this group, this proposed phylogeny will be a powerful tool for future studies.

## Supporting Information

Table S1 List of all individuals included in the phylogenetic analysis of Neoconocephalus. The position from the top in the AFLP tree (figure 1) is given, as well as the names used in the gene trees (figures 2–4). For US-localities we give the County, for other localities we give the town closest to the collection site. Country/State: CR Costa Rica, FL Florida (USA), MO Missouri (USA), NJ New Jersey (USA), PR Puerto Rico, TN Tennessee (USA), TT Trinidad and Tobago, TX Texas (USA). Collected by: DS D. Sattmann, JS J. Schul, KFH K.H. Frederick-Hudson, M.K. Brueggen, OMB O.M. Beckers, RLS R.L. Snyder, SLB S.L. Bush
(0.31 MB DOC)

## References

[1] Alexander RD (1956). A comparative study of sound production in insects with special reference to the singing Orthoptera and Cicadidae of the Eastern United States..

[2] Meixner AJ, Shaw KC (1986). Acoustic and associated behavior of the coneheaded katydid, *Neoconcocephalus nebrascensis* Orthoptera: Tettigoniidae.. Ann Entomol Soc Am.

[3] Greenfield MD (1983). Unsynchronized chorusing in the coneheaded katydid *Neoconocephalus affinis* (Beauvois).. Anim Behav.

[4] Brush JS, Gian VG, Greenfield MD (1985). Phonotaxis and aggression in the coneheaded katydid *Neoconocephalus affinis*.. Physiol Entomol.

[5] Meixner AJ, Shaw KC (1979). Spacing and movement of singing *Noeconocephalus nebrascensis* males (Orthoptera: Copiphorinae).. Ann Entomol Soc Am.

[6] Walker TJ (1975). Stridulatory movements in eight species of *Neoconocephalus* (Tettigoniidae).. J Insect Physiol.

[7] Heath JE, Josephson RK (1970). Body temperature and singing in the katydid *Neoconocephalus robustus* (Orthoptera, Tettigoniidae).. Biol Bull.

[8] Josephson, RK (1984). Contraction dynamics of flight and stridulatory muscles of tettigoniid insects.. J Exp Biol.

[9] Josephson RK (1985). The mechanical power output of a tettigoniid wing muscle during singing and flight.. J Exp Biol.

[10] Whitesell JJ, Walker TJ (1978). Photoperiodically determined dimorphic calling songs in a katydid.. Nature.

[11] Beckers OM, Schul J (2008). Developmental plasticity of mating calls enables acoustic communication in diverse environments.. Proc R Soc Lond B.

[12] Bush SL, Beckers OM, Schul J (2009). A complex mechanism of call recognition in the katydid *Neoconocephalus affinis* (Orthoptera: Tettigoniidae).. J Exp Biol.

[13] Deily JA, Schul J (2004). Recognition of calls with exceptionally fast pulse rates: Female phonotaxis in the genus *Neoconocephalus* (Orthoptera: Tettigoniidae).. J Exp Biol.

[14] Deily, JA, Schul J (2006). Spectral selectivity during phonotaxis: a comparative study in *Neoconocephalus* (Orthoptera, Tettigoniidae).. J Exp Biol.

[15] Deily JA, Schul J (2009). Selective Phonotaxis in *Neoconocephalus nebrascensis* (Orthoptera: Tettigoniidae): Call recognition at two temporal scales.. J Comp Physiol A.

[16] Greenfield MD, Roizen I (1993). Katydid synchronous chorusing is an evolutionarily stable outcome of female choice.. Nature.

[17] Greenfield, MD, Tourtellot, MK, Snedden, WA (1997). Precedence effects and the evolution of chorusing.. Proc R Soc Lond B.

[18] Greenfield MD, Bailey WJ, Rentz DCF (1990). Evolution of acoustic communication in the genus *Neoconocephalus*: Discontinuous songs, synchrony, and interspecific interactions.. The Tettigoniidae: Biology, Systematics and Evolution.

[19] Whitesell JJ (1969). Biology of United States coneheaded katydids of the genus *Neoconocephalus* (Orthoptera: Tettigoniidae)..

[20] Walker TJ, Whitesell JJ (1978). A new species of conehead from the Florida Everglades (Orthoptera: Tettigoniidae: *Neoconocephalus*).. Entomol News.

[21] Whitesell JJ (1974). Geographic variation and dimorphisms in song, development, and color in a katydid: field and laboratory studies (Tettigoniidae, Orthoptera)..

[22] Walker TJ, Greenfield MD (1983). Songs and systematics of Caribbean *Neoconocephalus* (Orthoptera, Tettigoniidae).. Trans Am Entomol Soc.

[23] Walker TJ, Whitesell JJ (1978). *Neoconocephalus maxillosus*: a Caribbean conehead in south Florida (Orthoptera: Tettigoniidae).. Fla Entomol.

[24] Walker TJ, Whitesell JJ, Alexander RD (1973). The robust conehead: two widespread sibling species (Orthoptera: Tettigoniidae: *Neoconocephalus ‘robustus’*).. Ohio J Sci.

[25] Walker TJ (2008). The Singing insects of North America – Katydids.. http://buzz.ifas.ufl.edu/crickets.htm.

[26] Eades DC, Otte D (2008). Orthoptera Species File Online - version 3.4.. http://Orthoptera.SpeciesFile.org.

[27] Redtenbacher J (1891). Monographie der Conocephaliden.. Verh Zool Bot Ges Wien.

[28] Rehn, JAG, Hebard M (1915). Studies in American Tettigoniidae (Orthoptera), III. Synopsis of the species of the genus *Neoconocephalus* found in North America north of Mexico.. Trans Am Entomol Soc.

[29] Bailey WJ, McCrae AWR (1978). The general biology and phenology of swarming in the East African tettigoniid *Ruspolia differens* (Serville) (Orthoptera).. J Nat Hist.

[30] Vos P, Hogers R, Bleeker M, Reijans M, Lee Tvd (1995). AFLP: A new technique for DNA fingerprinting.. Nucl Acids Res.

[31] Ji YJ, Zhang DX, He LJ (2003). Evolutionary conservation and versatility of a new set of primers for amplifying the ribosomal internal transcribed spacer regions in insects and other invertebrates.. Molecular Ecology Notes.

[32] Colgan DJ, Wilson GDF, Livingston SP, Edgecombe GD, Macaranas J (1998). Histone H3 and U2 Snrna DNA sequences and arthropod molecular evolution.. Austr J Zool.

[33] Lin CP, Wood TK (2002). Molecular phylogeny of the North American *Enchenopa binotata* (Homoptera: Membracidae) species complex.. Ann Entomol Soc Am.

[34] Nei M, Li WH (1979). Mathematical model for studying genetic variation in terms of restriction endonucleases.. Proc Nat Acad Sci USA.

[35] Swofford DL (2003). PAUP*. Phylogenetic analysis using parsimony (*and other methods)..

[36] Ronquist F, Huelsenbeck JP (2003). Mrbayes 3: Bayesian Phylogenetic Inference under Mixed Models.. Bioinformatics.

[37] Felsenstein J (1981). Evolutionary trees from DNA sequences: a maximum likelihood approach.. J Mol Evol.

[38] Koopman WJM, Wissemann V, De Cock K, Huylenbroeck JV, Riek JD (2008). AFLP markers as a tool to reconstruct complex relationships: A case study in *Rosa* (Rosaceae).. Am J Botany.

[39] Huelsenbeck JP, Bull JJ, Cunningham CW (1996). Combining data in phylogenetic analysis.. Trends Ecol Evol.

[40] Nylander J (2004). Mrmodeltest V2.0. distributed by author..

[41] Maddison DR, Maddison WP (2005). Macclade 4..

[42] Pagel M, Meade A (2007). Bayestraits.. http://www.evolution.rdg.ac.uk.

[43] Ballard JWO, Whitlock MC (2004). The incomplete natural history of mitochondria.. Mol Ecol.

[44] Mendelson TC, Shaw KL (2005). Sexual behaviour: Rapid speciation in an arthropod.. Nature.

[45] Schul J, Patterson AC (2003). What determines the tuning of hearing organs and the frequency of calls? A comparative study in the katydid genus *Neoconocephalus* (Orthoptera; Tettigoniidae).. J Exp Biol.

[46] Dobzhansky T (1937). Genetics and the origin of species..

[47] Nasrecki P (2000). Katydids of Costa Rica..

