# Supplementary material for: Molecular Phylogenetics of the Genus Neoconocephalus (Orthoptera, Tettigoniidae) and the Evolution of Temperate Life Histories
Source: PLoS One. 2009 Sep 25;4(9):e7203. doi: 10.1371/journal.pone.0007203 (PMC2745656; doi:10.1371/journal.pone.0007203)
Supplement: Table S1 — List of all individuals included in the phylogenetic analysis of Neoconocephalus. The position from the top in the AFLP tree (figure 1) is given, as well as the names used in the gene trees (figures 2– 4). For US-localities we give the County, for other localities we give the town closest to the collection site. Country/State: CR Costa Rica, FL Florida (USA), MO Missouri (USA), NJ New Jersey (USA), PR Puerto Rico, TN Tennessee (USA), TT Trinidad and Tobago, TX Texas (USA). Collected by: DS D. Sattmann, JS J. Schul, KFH K.H. Frederick-Hudson, M.K. Brueggen, OMB O.M. Beckers, RLS R.L. Snyder, SLB S.L. Bush (0.31 MB DOC) [file pone.0007203.s001.doc]

| Pos in AFLP tree | Name in trees | present in gene trees | species | sex | collection date | country / state | county / town | Collected by | Genbank code |
| --- | --- | --- | --- | --- | --- | --- | --- | --- | --- |
| 1 | Nro_MO-6 |  | *N. robustus* | m | 08/22/2006 | MO | Boone Co. | KFH | NB20 |
| 2 | Nro_MO-1 | x | *N. robustus* | m | 7/11/2003 | MO | Boone Co. | JS | NErobu |
| 3 | Nro_MO-2 | x | *N. robustus* | f | 8/24/2003 | MO | Boone Co. | JS | NErobu4 |
| 4 | Nro_MO-3 | x | *N. robustus* | f | 8/25/2003 | MO | Boone Co. | JS | NErobu3 |
| 5 | Nro_MO-4 |  | *N. robustus* | m | 08/14/2006 | MO | Boone Co. | KFH | NR3 |
| 6 | Nro_MO-5 | x | *N. robustus* | m | 08/2007 | MO | Boone Co. | KFH | NR15 |
| 7 | Nme_FL-1 |  | *N. melanorhinus* | m | 8/20/2003 | FL | Levy Co. | JS | NME |
| 8 | Nme_FL-2 | x | *N. melanorhinus* | m | 7/20/2005 | FL | Levy Co. | JS | NMECK3 |
| 9 | Nme_FL-3 | x | *N. melanorhinus* | m | 7/20/2005 | FL | Levy Co. | JS | NMECK1 |
| 10 | Nme_FL-4 |  | *N. melanorhinus* | m | 7/20/2005 | FL | Levy Co. | JS | NMECK2 |
| 11 | Nne_MO-2 |  | *N. nebrascensis* | m | 8/16/2006 | MO | Boone Co. | KFH | NN12 |
| 12 | Nne_MO-3 |  | *N. nebrascensis* | m | 8/29/2006 | MO | Boone Co. | KFH | NN16 |
| 13 | Nne_MO-1 | x | *N. nebrascensis* | m | 8/14/2006 | MO | Boone Co. | KFH | NN6 |
| 14 | Nne_MO-4 |  | *N. nebrascensis* | m | 08/2003 | MO | Boone Co. | JS | Nnebr |
| 15 | Nne_MO-5 | x | *N. nebrascensis* | m | 08/2005 | MO | Boone Co. | OMB | NnebrRT |
|  | Nne_MO-6 | x | *N. nebrascensis* | m | 8/14/2006 | MO | Boone Co. | KFH | NN7 |
| 16 | Nbi_MO-3 | x | *N. bivocatus* | m | 08/16/2006 | MO | Boone Co. | KFH | NB11 |
| 17 | Nbi_MO-2 | x | *N. bivocatus* | m | 07/2001 | MO | Boone Co. | JS | NBivo |
| 18 | Nbi_MO-1 | x | *N. bivocatus* | f | 8/24/2003 | MO | Boone Co. | JS | NBivo2 |
| 19 | Nex_MO-4 |  | *N. exciliscanorus* | m | 7/23/2006 | MO | Stoddard Co. | JS | NexMO1 |
| 20 | Nex_MO-5 | x | *N. exciliscanorus* | m | 7/23/2006 | MO | Stoddard Co. | JS | NexMO2 |
| 21 | Nex_MO-1 | x | *N. exciliscanorus* | m | 8/16/2007 | MO | Stoddard Co. | JS/RLS | NExMO3 |
| 22 | Nex_MO-2 | x | *N. exciliscanorus* | m | 8/16/2007 | MO | Stoddard Co. | JS/RLS | NExMO4 |
| 23 | Nex_MO-3 |  | *N. exciliscanorus* | m | 8/16/2007 | MO | Stoddard Co. | JS/RLS | NExMO5 |
| 24 | Nex_NJ-1 | x | *N. exciliscanorus* | m | 9/4/2007 | NJ | Atlantic Co. | KFH | NExNJ1 |
| 25 | Nex_NJ-2 | x | *N. exciliscanorus* | m | 9/4/2007 | NJ | Atlantic Co. | KFH | NExNJ3 |
| 26 | Nex_NJ-3 | x | *N. exciliscanorus* | m | 9/4/2007 | NJ | Atlantic Co. | KFH | NExNJ2 |
| 27 | Nca_FL-1 | x | *N. caudellianus* | m | 7/21/2005 | Fl | Alachua Co. | JS/OMB | NCGV2 |
| 28 | Nca_FL-2 | x | *N. caudellianus* | m | 7/21/2005 | Fl | Alachua Co. | JS/OMB | NCGV3 |
| 29 | Nca_FL-3 |  | *N. caudellianus* | m | 7/21/2005 | Fl | Alachua Co. | JS/OMB | NCGV4 |
| 30 | Nen_MO-1 | x | *N. ensiger* | m | 7/11/2003 | MO | Boone Co. | JS | NE |
| 31 | Nen_MO-2 | x | *N. ensiger* | m | 7/11/2003 | MO | Boone Co. | JS | NE3 |
| 32 | Nen_MO-3 |  | *N. ensiger* | m | 08/27/2007 | MO | Adair Co. | KFH | NEMOkv1 |
| 33 | Nen_MO-4 | x | *N. ensiger* | m | 08/27/2007 | MO | Adair Co. | KFH | NEMOkv2 |
| 34 | Nen_MO-5 | x | *N. ensiger* | m | 08/27/2007 | MO | Adair Co. | KFH | NEMOkv3 |
| 35 | Ntr_MO-1 | x | *N. triops* | m | 6/28/2003 | MO | Boone Co. | JS | NEtrio |
| 36 | Ntr_FL-1 |  | *N. triops* | m | 8/19/2003 | FL | Alachua Co. | JS/OMB | NEtrio4 |
| 37 | Ntr_TN-3 |  | *N. triops* | m | 9/19/2006 | TN | Dyer Co. | JS | NTTN3 |
| 38 | Ntr_FL-2 | x | *N. triops* | m | 8/19/2003 | FL | Alachua Co. | JS/OMB | NEtrio3 |
| 39 | Ntr_FL-3 |  | *N. triops* | f | 8/19/2003 | FL | Alachua Co. | JS/OMB | NEtrio2 |
| 40 | Ntr_TN-1 |  | *N. triops* | f | 9/15/2006 | TN | Dyer Co. | JS | NtrioTN1 |
| 41 | Ntr_TN-2 |  | *N. triops* | f | 9/16/2006 | TN | Dyer Co. | JS | NtrioTN2 |
| 42 | Ntr_FL-4 |  | *N. triops* | m | 3/3/2004 | FL | Alachua Co. | OMB | NEtrio5 |
| 43 | Ntr_PR-1 | x | *N. triops* | m | 1/6/2006 | PR | Naguabo | SLB | NEtrioPR1 |
| 44 | Ntr_PR-2 | x | *N. triops* | m | 1/7/2006 | PR | Lafe | SLB | NEtrioPR3 |
| 45 | Ntr_PR-3 |  | *N. triops* | m | 1/7/2006 | PR | Humaco | SLB | NEtrioPR2 |
| 46 | Ntr_CR-1 |  | *N. triops* | m | 3/28/2007 | CR | Bagaces | OMB/SLB | NEtrioCR1 |
| 47 | Ntr_CR-2 |  | *N. triops* | m | 3/28/2007 | CR | Bagaces | OMB/SLB | NEtrioCR3 |
| 48 | Ntr_CR-3 | x | *N. triops* | m | 3/28/2007 | CR | Bagaces | OMB/SLB | NEtrioCR2 |
| 49 | Ntr_CR-4 | x | *N. triops* | m | 3/28/2007 | CR | Bagaces | OMB/SLB | NEtrioCR4 |
| 50 | Ntr_CR-5 |  | *N. triops* | m | 3/28/2007 | CR | Bagaces | OMB/SLB | NEtrioCR5 |
| 51 | Ntr_TT-1 |  | *N. triops* | m | 9/28/2007 | TT | Arima | RLS/JS | NtTT2 |
| 52 | Ntr_TT-3 | x | *N. triops* | m | 9/29/2007 | TT | Piarco | RLS/JS | NtTT4 |
| 53 | Ntr_TT-4 | x | *N. triops* | m | 9/29/2007 | TT | Piarco | RLS/JS | NtTT5 |
| 54 | Ntr_TT-2 |  | *N. triops* | m | 9/28/2007 | TT | Arima | RLS/JS | NtTT3 |
| 55 | Ntr_TT-5 |  | *N. triops* | m | 9/29/2007 | TT | Piarco | RLS/JS | NtTT6 |
| 56 | Ntr_TN-4 |  | *N. triops* | m | 4/30/2007 | TN | Dyer Co. | OMB | NtTN4 |
| 57 | Ntr_TX-1 | x | *N. triops* | m | 8/1/2007 | TX | Travis Co. | DS | NtTX2 |
| 58 | Ntr_TX-2 |  | *N. triops* | m | 8/1/2007 | TX | Travis Co. | DS | NtTx5 |
| 59 | Ntr_TN-5 |  | *N. triops* | m | 4/30/2007 | TN | Dyer Co. | OMB | NtTN5 |
| 60 | Ntr_TX-3 |  | *N. triops* | m | 8/1/2007 | TX | Travis Co. | DS | NtTx4 |
| 61 | Ntr_TX-4 | x | *N. triops* | m | 8/1/2007 | TX | Travis Co. | DS | NtTx3 |
| 62 | Ntr_TX-5 |  | *N. triops* | m | 8/1/2007 | TX | Travis Co. | DS | NtTx1 |
| 63 | Nsp_CR-1 | x | *N. spiza* | m | 3/27/2007 | CR | Tilaran | OMB/SLB | NSCR3 |
| 64 | Nsp_CR-2 | x | *N. spiza* | m | 3/27/2007 | CR | Tilaran | OMB/SLB | NSCR1 |
| 65 | Nsp_CR-3 |  | *N. spiza* | m | 3/27/2007 | CR | Tilaran | OMB/SLB | NSCR5 |
| 66 | Nsp_CR-4 | x | *N. spiza* | m | 3/27/2007 | CR | Tilaran | OMB/SLB | NSCR2 |
| 67 | Nsp_CR-5 |  | *N. spiza* | m | 3/27/2007 | CR | Tilaran | OMB/SLB | NSCR4 |
| 68 | Nma_PR-1 |  | *N. maxillosus* | m | 1/6/2006 | PR | Naguabo | SLB | NMAPR1 |
| 69 | Nma_PR-4 |  | *N. maxillosus* | m | 1/9/2006 | PR | Naguabo | SLB | NMAPR4 |
| 70 | Nma_PR-2 | x | *N. maxillosus* | m | 1/7/2006 | PR | Naguabo | SLB | NMAPR2 |
| 71 | Nma_PR-3 |  | *N. maxillosus* | m | 1/7/2006 | PR | Lafe | SLB | NMAPR3 |
| 72 | Nma_PR-5 | x | *N. maxillosus* | f | 1/8/2006 | PR | Lafe | SLB | NMaPR5 |
|  | Nma_PR-6 | x | *N. maxillosus* | F | 3/1/2007 | PR | Naguabo | SLB | NmaPR6 |
| 73 | Nma_TT-1 | x | *N. maxillosus* | m | 9/28/2007 | TT | Arima | RLS/JS | NmaTT1 |
| 74 | Nma_TT-2 |  | *N. maxillosus* | m | 9/28/2007 | TT | Arima | RLS/JS | NmaTT2 |
| 75 | Nma_TT-3 |  | *N. maxillosus* | m | 9/28/2007 | TT | Piarco | RLS/JS | NmaTT3 |
| 76 | Nma_TT-4 | x | *N. maxillosus* | m | 9/29/2007 | TT | Arima | RLS/JS | NmaTT4 |
| 77 | Nsa_TT-7 |  | *N. saturatus* | m | 9/29/2007 | TT | Piarco | RLS/JS | NsaTT7 |
| 78 | Nsa_TT-5 | x | *N. saturatus* | m | 9/28/2007 | TT | Piarco | RLS/JS | NsaTT5 |
| 79 | Nsa_TT-6 | x | *N. saturatus* | m | 9/29/2007 | TT | Piarco | RLS/JS | NsaTT6 |
| 80 | Nsa_TT-1 | x | *N. saturatus* | m | 9/28/2007 | TT | Arima | RLS/JS | NsaTT1 |
| 81 | Nsa_TT-4 |  | *N. saturatus* | m | 9/28/2007 | TT | Piarco | RLS/JS | NsaTT4 |
| 82 | Npa_MO-1 | x | *N. palustris* | m | 8/16/2007 | MO | Stoddard Co. | RLS/JS | NPaMO1 |
| 83 | Npa_MO-2 | x | *N. palustris* | m | 8/16/2007 | MO | Stoddard Co. | RLS/JS | NPaMO2 |
| 84 | Npa_TN-1 | x | *N. palustris* | f | 9/17/2006 | TN | Dyer Co. | JS/MKB | NEpalTN1 |
| 85 | Npa_TN-2 | x | *N. palustris* | f | 9/18/2006 | TN | Dyer Co. | JS/MKB | NEpalTN2 |
| 86 | Nve_FL-1 | x | *N. velox* | m | 7/20/2005 | FL | Levy Co. | OMB/JS | NEveCK1 |
| 87 | Nve_FL-2 |  | *N. velox* | m | 7/20/2005 | FL | Levy Co. | OMB/JS | NEveCK2 |
| 88 | Nve_FL-3 |  | *N. velox* | m | 7/20/2005 | FL | Alachua Co. | OMB/JS | NEveGV1 |
| 89 | Nve_FL-4 | x | *N. velox* | m | 7/21/2005 | Fl | Alachua Co. | OMB/JS | NEveGV2 |
| 90 | Nrf_PR-1 | x | *N. retusiformis* | m | 1/9/2006 | PR | Naguabo | OMB/JS | NRFPR1 |
| 91 | Naf_TT-2 |  | *N. affinis* | m | 9/29/2007 | TT | Arima | RLS/JS | NaTT6 |
| 92 | Naf_TT-3 |  | *N. affinis* | m | 9/29/2007 | TT | Arima | RLS/JS | NaTT7 |
| 93 | Naf_TT-4 |  | *N. affinis* | m | 9/29/2007 | TT | Arima | RLS/JS | NaTT5 |
| 94 | Naf_TT-5 | x | *N. affinis* | m | 9/29/2007 | TT | Arima | RLS/JS | NaTT4 |
| 95 | Naf_TT-1 | x | *N. affinis* | m | 9/28/2007 | TT | Arima | RLS/JS | NaTT1 |
| 96 | Naf_PR-1 | x | *N. affinis* | m | 1/6/2006 | PR | Naguabo | OMB/JS | NAPR2 |
| 97 | Naf_PR-2 | x | *N. affinis* | m | 1/6/2006 | PR | Naguabo | OMB/JS | NAPR3 |
| 98 | Naf_PR-3 |  | *N. affinis* | m | 1/6/2006 | PR | Naguabo | OMB/JS | NAPR1 |
| 99 | Naf_PR-5 |  | *N. affinis* | m | 1/3/2007 | PR | Lafe | OMB/JS | NAPR5 |
| 100 | Naf_PR-4 |  | *N. affinis* | m | 1/12/2006 | PR | Luquillo | OMB/JS | NAPR4 |
| 101 | Naf_CR-1 |  | *N. affinis* | m | 3/27/2007 | CR | Tilaran | SLB/OMB | NACR5 |
| 102 | Naf_CR-2 |  | *N. affinis* | m | 3/27/2007 | CR | Tilaran | SLB/OMB | NACR4 |
| 103 | Naf_CR-3 |  | *N. affinis* | m | 3/27/2007 | CR | Tilaran | SLB/OMB | NACR3 |
| 104 | Naf_CR-4 | x | *N. affinis* | m | 3/27/2007 | CR | Tilaran | SLB/OMB | NACR2 |
| 105 | Naf_CR-5 | x | *N. affinis* | m | 3/27/2007 | CR | Tilaran | SLB/OMB | NACR1 |
| 106 | Nre_MO-1 | x | *N. retusus* | m | 09/2002 | MO | Boone Co. | JS | NRE2 |
| 107 | Nre_FL-1 |  | *N. retusus* | m | 8/19/2003 | FL | Alachua Co. | JS/OMB | NRE4 |
| 108 | Nre_MO-2 |  | *N. retusus* | m | 8/25/2003 | MO | Boone Co. | JS | NRE7 |
| 109 | Nre_TN-4 | x | *N. retusus* | m | 9/12/2006 | TN | Dyer Co. | JS/MKB | NRETN1 |
| 110 | Nre_TN-5 | x | *N. retusus* | m | 9/13/2006 | TN | Dyer Co. | JS/MKB | NRETN2 |
|  | Nre_FL-2 | x | *N. retusus* | m | 08/19/03 | FL | Alachua Co. | JS/OMB | NRE5 |
| 111 | Npu_CR-1 |  | *N. punctipes* | m | 3/26/2007 | CR | Bagaces | SLB/OMB | NPUCR1 |
| 112 | Npu_CR-2 | x | *N. punctipes* | m | 3/26/2007 | CR | Bagaces | SLB/OMB | NPUCR2 |
| 113 | Npu_CR-3 | x | *N. punctipes* | m | 3/27/2007 | CR | Tilaran | SLB/OMB | NPUCR3 |
| 114 | Npu_CR-4 |  | *N. punctipes* | m | 3/27/2007 | CR | Tilaran | SLB/OMB | NPUCR4 |
| 115 | Npu_CR-5 |  | *N. punctipes* | m | 3/27/2007 | CR | Tilaran | SLB/OMB | NPUCR5 |
| 116 | Npu_TT-1 | x | *N. punctipes* | m | 9/29/2007 | TT | Piarco | RLS/JS | NpuTT3 |
| 117 | Npu_TT-2 |  | *N. punctipes* | m | 9/29/2007 | TT | Piarco | RLS/JS | NpuTT5 |
| 118 | Npu_TT-3 |  | *N. punctipes* | m | 9/29/2007 | TT | Piarco | RLS/JS | NpuTT4 |
| 119 | Npu_TT-4 | x | *N. punctipes* | m | 9/29/2007 | TT | Piarco | RLS/JS | NpuTT2 |
| 120 | Bma_FL-5 |  | *Bucrates malivolans* | m | 7/20/2005 | FL | Levy Co. | JS/OMB | BuMCK1 |
| 121 | Bma_FL-1 | x | *Bucrates malivolans* | m | 8/20/2003 | FL | Levy Co. | JS/OMB | BUmali2 |
| 122 | Bma_FL-2 | x | *Bucrates malivolans* | m | 8/20/2003 | FL | Levy Co. | JS/OMB | BUmali3 |
| 123 | Bma_FL-3 |  | *Bucrates malivolans* | m | 8/20/2003 | FL | Levy Co. | JS/OMB | BUmali4 |
| 124 | Bma_FL-4 |  | *Bucrates malivolans* | m | 8/20/2003 | FL | Levy Co. | JS/OMB | Bumali |
|  | Bda_FL-1 | x | *Belocephalus davisi* | m | 08/20/03 | FL | Levy Co. | JS/OMB | Bedavi3 |
